# Predicting Multiple Traits of Rice and Cotton Across Varieties and Regions Using Multi-Source Data and a Meta-Hybrid Regression Ensemble

**DOI:** 10.3390/s26020375

**Published:** 2026-01-06

**Authors:** Yu Qin, Moughal Tauqir, Xiang Yu, Xin Zheng, Xin Jiang, Nuo Xu, Jiahua Zhang

**Affiliations:** 1 Remote Sensing Information and Digital Earth Center, College of Computer Science and Technology, Qingdao University, Qingdao 266071, China; qinyu@qdu.edu.cn (Y.Q.); 2020020669@qdu.edu.cn (M.T.); 2020010031@qdu.edu.cn (X.Y.); 2021020707@qdu.edu.cn (X.Z.); jiangxin1@qdu.edu.cn (X.J.); 2 Aerospace Information Research Institute, Chinese Academy of Sciences, Beijing 100094, China; 3 Department of Biological and Agriculture Engineering, University of California, Davis, CA 95616, USA; nnnxu@ucdavis.edu

**Keywords:** crop traits prediction, crop varieties, Meta-Hybrid Regression Ensemble, multi-source data fusion, machine learning, remote sensing

## Abstract

Timely and accurate prediction of crop traits is critical for precision breeding and regional agricultural production. Previous studies have primarily focused on single crop yield traits, neglecting other crop traits and variety-specific analyses. To address this issue, we employed a Meta-Hybrid Regression Ensemble (MHRE) approach by using multiple machine learning (ML) approaches as base learners, integrating regional multi-year, multi-variety crop field trials with satellite remote sensing indices, meteorological and phenological data to predict major crop traits. Results demonstrated MHRE’s optimal performance for rice and cotton, significantly outperforming individual models (RF, XGBoost, CatBoost, and LightGBM). Specifically, for rice crop, MHRE achieved highest accuracy for yield trait (R^2^ = 0.78, RMSE = 0.59 t ha^−1^) compared to the best individual model (XGBoost: R^2^ = 0.76, RMSE = 0.61 t ha^−1^); traits like effective spike also showed strong predictability (R^2^ = 0.64, RMSE = 27.81 10,000·spike ha^−1^). Similarly, for cotton, MHRE substantially improved yield trait prediction (R^2^ = 0.82, RMSE = 0.33 t ha^−1^) compared to the best individual model (RF: R^2^ = 0.77, RMSE = 0.36 t ha^−1^); bolls per plant accuracy was highest (R^2^ = 0.93, RMSE = 2.27 bolls plant^−1^). Moreover, rigorous validation confirmed that crop-specific MHRE models are robust across five rice and three cotton varietal groups and are applicable across six distinct regions in China. Furthermore, we applied the SHAP (SHapley Additive exPlanations) method to analyze the growth stages and key environmental factors affecting major traits. Our study illustrates a practical framework for regional-scale crop traits prediction by fusing multi-source data and ensemble machine learning, offering new insights for precision agriculture and crop management.

## 1. Introduction

By 2050, the global population is projected to increase by more than two billion [1]. To sustainably secure food supplies under this mounting pressure, it is imperative to elucidate the evolutionary regulatory mechanisms underlying key crop traits and to translate fundamental discoveries into breeding practice [2,3]. Based on the precise prediction of crop traits, we can not only establish a molecular design breeding system to accelerate the development and screening of new cultivars [4,5], but also optimize the spatiotemporal allocation of field resources and provide scientific support for formulating smart agriculture policies [6,7]. Moreover, by integrating trait-prediction data, growers and agronomists can develop dynamic decision-support models for finely tuned crop management [8].

In agricultural production, crop yield serves as a critical indicator of productivity [9]. Although yield is an important indicator, it represents the cumulative outcome of multiple complex biological processes, making it difficult to capture the real-time dynamics of crop growth. In contrast, key crop traits—such as thousand-kernel weight, stem height, and photosynthetic efficiency—are essential for characterizing crop physiological status and potential productivity [10,11,12]. These traits can reflect crop physiological states and stress responses at enhanced spatial and temporal resolutions [13]. Recent advancements in multispectral/hyperspectral remote sensing technologies have substantially improved the precision of crop traits prediction [14,15]. However, existing research predominantly focuses on end-season yield prediction [16], while systematic predictive modeling of these critical crop traits remains relatively underdeveloped [17]. Furthermore, most current findings are constrained to single cultivars or specific regions, with their robustness and reliability across diverse varieties and geographical areas yet to be thoroughly validated [18]. Therefore, in-depth research on predicting key traits of major crops is crucial for enhancing agricultural production efficiency and refining field management decisions.

Satellite remote sensing, particularly using multispectral bands, provides powerful tools for monitoring crop growth and characterizing field spatial heterogeneity [19]. Vegetation indices (VIs) derived from visible and near-infrared bands effectively reflect photosynthetic activity and biomass dynamics [20,21]. With advances in remote sensing technology, an increasing number of novel spectral products are being incorporated into crop characterization research. For instance, sun-induced chlorophyll fluorescence (SIF), originating from specific narrow bands in the near-infrared region, provides a more precise proxy indicator for instantaneous crop photosynthetic efficiency [22,23], while the retrieval of gross primary productivity (GPP) directly reflects the net photosynthetic rate of crops [24,25]. Additionally, soil moisture products derived from microwave sensors such as SMAP and ASCAT can be utilized to assess surface soil moisture conditions and root zone water availability [26]. Particularly, meteorological factors (e.g., air temperature, precipitation) profoundly influence crop physiology [27,28], yet spectral signals alone often fail to fully capture these abiotic drivers, necessitating integration with ground-based or reanalysis data [29]. Similarly, anthropogenic factors such as field management practices significantly impact crop traits, yet these are typically not directly measurable via satellite remote sensing [30,31]. While data from various satellite platforms are extensively applied to evaluate crop growth status, research integrating remote sensing data, meteorological data, and field data for comprehensive crop trait prediction remains relatively scarce.

Currently, three primary approaches are employed for predicting crop traits. The first approach involves process-based growth simulation combined with data assimilation [32]. This method simulates crop growth and development by finely tuning model parameters to generate predictions [33]. However, the complexity of model parameters and the stringent requirements for initial conditions limit its efficiency and scalability for large-scale applications [34,35]. The second approach utilizes statistical regression models to establish relationships between vegetation indices and crop traits. Nevertheless, this method exhibits limited capability in modeling nonlinear relationships and suffers from inadequate generalization across spatial and temporal scales [36]. The third approach is based on machine learning (ML) algorithms. Traditional ML methods, such as Random Forest (RF) and Extreme Gradient Boosting (XGBoost), have been widely applied in crop traits prediction [37,38,39]. However, these methods often struggle to effectively capture the complex interactions between crop traits and environmental factors and face challenges in integrating heterogeneous data. Moreover, constraints in data quantity and quality have confined most studies to single-yield predictions at county or municipal levels [40,41]. As a branch of ML, deep learning (DL) excels in agricultural remote sensing tasks thanks to its powerful feature extraction capabilities. Algorithms such as convolutional neural networks [42,43] have demonstrated remarkable effectiveness in field-scale crop phenotyping using high-resolution satellite data. However, the reliability of these models critically depends on the quantity and quality of field-collected samples. In China’s agricultural sector, the scarcity of such samples and the high cost of their acquisition have limited the large-scale deployment of DL [44].

Despite these advances, current studies on crop trait prediction still face several key limitations. Most work has focused on end-of-season yield rather than on multiple crop traits at regional scales, and is often restricted to single cultivars or limited regions without rigorous evaluation across varietal groups and ecological zones. In addition, the joint use of multi-year, multi-variety field trials with multi-source satellite products and meteorological variables remains uncommon, and existing modelling efforts typically optimise a single algorithm rather than exploiting the complementary strengths of multiple machine learning methods within an ensemble and meta-learning framework. Together, these limitations highlight the need for a general yet practical framework that can perform multi-trait prediction for major crops, make efficient use of heterogeneous data sources, and generalise robustly across varieties and regions.

To overcome these issues, achieving spatial and temporal generalization, performing multi-trait joint prediction, and reducing data sample dependence, we apply a Meta-Hybrid Regression Ensemble (MHRE) approach, using Linear_regression, XGBoost, Decision_tree, RF, LightGBM, and CatBoost as base learners combined with meta-learning strategies and ensemble learning techniques for crop multi-trait prediction. MHRE integrates multiple regression algorithms with a meta-learning strategy [45], thereby overcoming the tendency of single models to become trapped in local optima and their inability to generalize across regions and varieties [46,47]. Furthermore, we incorporate meteorological, remote sensing data, and publicly available field trial data to quantitatively analyze yield drivers [48]. In this study, the MHRE framework is applied in a crop-specific manner, with separate models trained for rice and for cotton using their respective multi-year regional field trial datasets.

The main objectives of this study are: (1) to integrate multi-source data and finely characterize crop growth environments using remote sensing and meteorological features; (2) to construct a scalable MHRE approach that outperforms traditional algorithms in regression prediction tasks; (3) to predict major traits of rice and cotton using crop-specific MHRE models across multiple varietal groups and regions in China, and to evaluate the robustness of the framework within each crop; and (4) to use the SHapley Additive Explanations (SHAP) method to improve model interpretability by identifying and quantifying key growth stages and critical environmental factors.

## 2. Materials and Methods

### 2.1. Study Area

**Figure 1:**
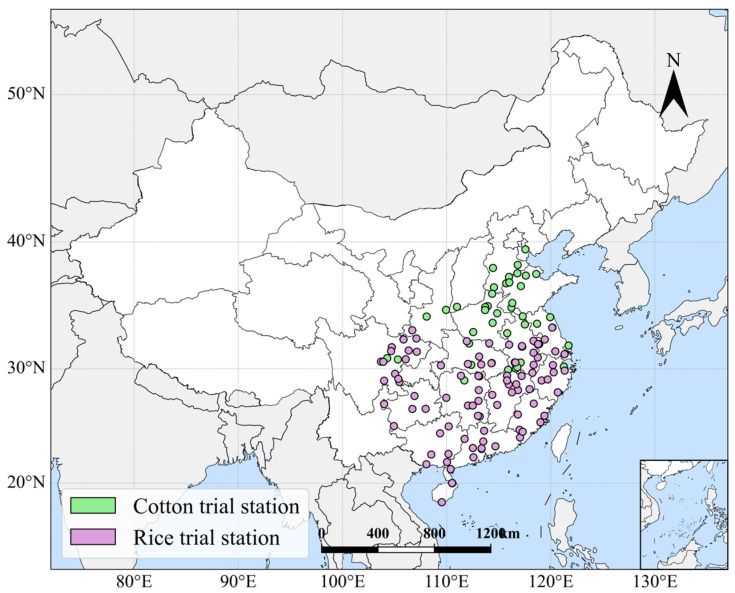
Study area showing the spatial distribution of 94 rice and 45 cotton stations in China, where green represents cotton and purple represents rice.

The sites shown in Figure 1 encompass most of the experimental fields for the national and regional trials of rice and cotton. The cotton trial stations (green markers) are located in the Yellow River and Yangtze River Basins, which are characterized by favorable climatic conditions and abundant agricultural resources, making them the most important cotton production bases in China.

The rice trial stations (purple markers) are primarily situated in the eastern and southwestern regions of China, focusing on core production areas such as the middle and lower plains of the Yangtze River and the Pearl River Delta, which have the most suitable agro-ecological conditions for rice cultivation and support a high-yield and high-quality rice production system.

### 2.2. Data Sources

#### 2.2.1. Remote Sensing Data

As shown in Table 1, this study used the National Aeronautics and Space Administration’s (NASA) Moderate Resolution Imaging Spectroradiometer (MODIS) remote sensing dataset and the Solar Induced Chlorophyll Fluorescence (SIF) dataset acquired by the TROPOMI sensor on board ESA’s Sentinel-5P satellite. The following MODIS products were used in this study: Normalized Difference Vegetation Index (NDVI), Leaf Area Index (LAI), Potential Evapotranspiration (PET), Evapotranspiration (ET), Gross Primary Productivity (GPP), Fraction of Absorbed Photosynthetic Active Radiation (Fpar). Moreover, the Chinese 1 km resolution daily all-weather surface soil moisture dataset from the National Tibetan Plateau Science Data Center [49] was used. The 36 km-resolution soil moisture (SM) data from AMSR-E and AMSR-2 were integrated with MODIS reflectance and land surface temperature (LST) using a downscaling model to form a high-coverage soil moisture product at 1 km resolution. To ensure spatial consistency among the multi-source datasets with different native spatial resolutions (500 m to ~3.5 km), all remote sensing variables were expressed at a common 1 km spatial resolution. For the 500 m MODIS products, 1 km values were obtained by aggregating each 2 × 2 block of original pixels using the arithmetic mean. For the coarser TROPOMI SIF data (~0.05°, ~3.5 km), 1 km values at the MODIS locations were derived by bilinear interpolation from the original grid. The 1-km soil-moisture product was reprojected to the same coordinate system while maintaining its native resolution. Field plots from the regional variety trials were geolocated in this reference system and used to extract the corresponding 1-km predictors from each dataset. All reprojection and resampling operations were implemented using standard GIS tools (e.g., GDAL, rasterio, xarray) to ensure consistent spatial alignment across sensors and years.

In addition, we used a national 1 km land-use remote-sensing dataset provided by the Resources and Environmental Science and Data Center of the Chinese Academy of Sciences to distinguish cropland from non-cropland areas and to reduce contamination of vegetation indices by surrounding roads, buildings, water bodies and forests. In this product, cultivated land is clearly separated from water bodies, urban land, residential land and other non-agricultural types. After aligning the land-use map with the remote-sensing imagery, we applied a cropland mask: NDVI, LAI and other remote-sensing predictors were extracted only from pixels classified as cultivated land, while pixels mapped as non-cropland were discarded and regional variety trial plots falling in such pixels were excluded from the modelling dataset. For each remaining plot, local canopy conditions were then represented by averaging the values of cropland pixels within a 2 × 2 neighbourhood of 1 km pixels around the plot location, which further mitigates mixed-pixel effects near field boundaries at this spatial resolution.

#### 2.2.2. Meteorological Data

The meteorological data used in this study were obtained from the European Center for Medium-Range Weather Forecasts (ECMWF) ERA5-Land reanalysis products covering the period from 2006 to 2023 with a spatial resolution of 0.1° and a temporal resolution of daily values. The main meteorological variables extracted included daily mean air temperature, daily maximum air temperature, daily minimum air temperature, and 2 m dew point temperature, from which four key environmental indicators were calculated: growing degree days (GDD, Equation (1), °C${\mathrm{days}}^{-1}$), saturated water vapor pressure difference (VPD, Equation (2), hPa), killing degree days (KDD, Equation (3), °C${\mathrm{days}}^{-1}$), and cumulative precipitation (Prcp, Equation (4), mm), which were used to characterize heat stress, water stress, heat accumulation, and precipitation supply during the growing period of the crop, respectively. Their calculation formulas are as follows:

(1) $$\mathrm{GDD}=\sum_{d=1}^{N}\left(\frac{T_{\max}^{d}+T_{\min}^{d}}{2}-T_{\mathrm{base}}\right)$$

(2) $$\mathrm{VPD}=\sum_{d=1}^{N}\left(e_{s}-e_{a}\right)$$

(3) $$\mathrm{KDD}=\sum_{d=1}^{N}\max\left(T_{\max}^{d}-29,0\right)$$

(4) $$\mathrm{Prep}=\sum_{d=1}^{N}{\mathrm{Prep}}^{d}$$

where N is the number of days in the growth period; $T_{\max}^{d}$ and $T_{\min}^{d}$ represent the daily maximum and minimum temperatures (°C) on day d, respectively; the base temperature $T_{\mathrm{base}}$ is set at 10 °C for rice and 12 °C for cotton; $P{\mathrm{rec}}^{d}$ denotes the daily precipitation (mm) on day d; $e_{s}$ is the saturated vapor pressure, and is the actual vapor pressure.

#### 2.2.3. Experimental Field Data

The experimental field data for this study were obtained from the National Variety Regional Trial Network of the Ministry of Agriculture and Rural Affairs of China (MARD), which integrates a multi-cycle regional trial dataset for rice from 2006 to 2016, and trial data for cotton obtained over seven non-consecutive years within the period from 2008 to 2023. In total, the compiled dataset includes 13,998 rice samples from 94 trial stations and 8046 cotton samples from 45 stations. These experimental fields were established in typical rice and cotton growing areas to represent different climatic conditions in the region. All data were collected following standardized operating procedures. Data underwent quality checks after data entry to identify and remove anomalies and to make necessary statistical corrections. These detailed experimental field data provide information on crop responses under real environmental conditions and support in-depth studies on the physiological and yield responses of rice and cotton. Table 2 below shows the main parameters of the experimental field data for rice and cotton.

### 2.3. Modeling Framework

**Figure 2:**
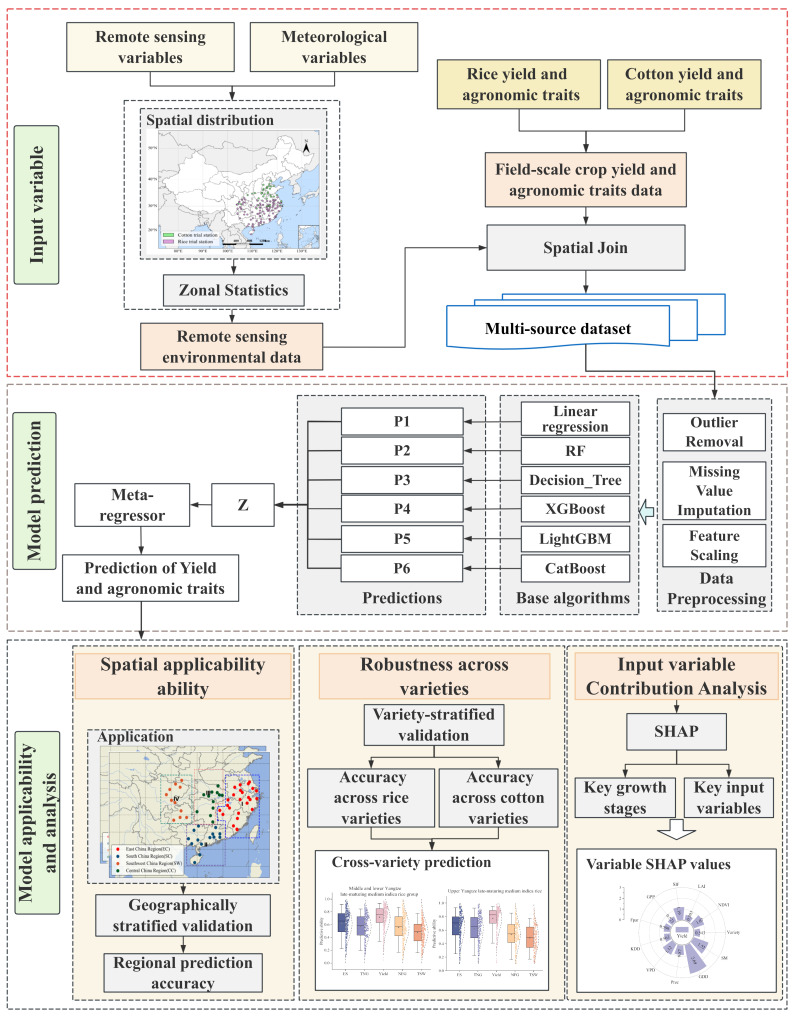
Overall methodological flowchart for major crop traits prediction of rice and cotton through multi-source data. P represents the predictions generated by the base model.

In this study, we developed an MHRE framework that integrates multiple regression algorithms with meta-learning strategies and ensemble learning techniques for the prediction of major crop traits. The framework integrates multi-source remote sensing data (NDVI, LAI, GPP, SM, SIF, Fpar, ET, and PET), meteorological variables (VPD, GDD, Prec, and KDD), and phenological records and major crop traits from multi-year field trials (Figure 2). The agronomic data collected in the field and the pre-processed meteorological parameters were fused with remote sensing indices after systematic quality control and unified coding to form a complete multi-source database. For both rice and cotton, plot-level phenological observations from the regional variety trials were used to align meteorological and remote-sensing variables across years and cycles. Rather than using raw calendar dates, NDVI, LAI and other satellite-derived indices were reindexed relative to key phenological milestones. For rice, sowing, heading and maturity dates defined two main growth phases (sowing–heading and heading–maturity), within which remote-sensing variables were sampled at 10-day intervals for each plot-year. For cotton, an analogous procedure was applied using seeding, flowering and batting dates. For both crops, cumulative GDD, KDD, precipitation and VPD were computed over these phenology-based windows, providing stage-specific summaries of thermal and hydroclimatic conditions. To mitigate the effects of multicollinearity and enhance model generalizability, a recursive feature elimination (RFE) method was employed to pre-select the most informative predictors from the initial feature set. This process identified and retained a subset of non-redundant features that contributed most significantly to the predictive performance, which were then used as inputs for all subsequent machine learning models. The database was divided into 70% training set and 30% test set to ensure the independence of evaluation. To reduce the impact of measurement errors and extreme values, we applied IQR- and Z-score-based outlier filtering, mean imputation and standardization to the multi-year, multi-cycle trial data. Generalization was evaluated using geographically and varietally stratified cross-validation, yielding a more realistic estimate of predictive performance under heterogeneous conditions.

In this study, the MHRE framework was applied separately to rice and cotton, with each crop model trained on its own multi-year field trial dataset. These four tree-based ensemble methods (RF, XGBoost, LightGBM and CatBoost) were selected because they are widely used and well validated for tabular environmental and remote-sensing data, can effectively capture nonlinear relationships and high-order interactions between crop traits and predictors, and represent complementary algorithmic families (bagging versus gradient boosting), which increases model diversity and improves the performance of the meta-ensemble. The inclusion of linear regression alongside tree-based learners introduces heterogeneity in inductive biases within the ensemble. Tree-based models are well-suited for capturing complex nonlinear interactions and hierarchical feature dependencies, whereas linear regression provides an efficient mechanism for modeling global linear trends. Such diversity is a core principle of ensemble learning, as it allows the meta-learner to synergistically combine the distinct strengths of each model class. The hyper-parameter optimization was carried out by grid search in combination with 5-fold cross-validation. Model performance is quantified based on coefficient of determination (R^2^), Root Mean Square Error (RMSE), Root Relative Mean Squared Error (RRMSE), Ratio of Performance to Deviation (RPD), and Mean Absolute Error (MAE) metrics of the test set, complemented by residual analysis to verify predictive stability. Among the base learners, relatively more complex ML models including XGBoost, RF, LightGBM and CatBoost were selected as baseline models for comparison with the MHRE framework. The generalization ability of the framework is verified through spatial domain cross-validation and new variety testing, while the relative contribution of remote sensing and meteorological variables is elucidated using built-in feature importance indicators and SHAP value analysis to achieve model interpretability.

### 2.4. Regression Algorithm

**Figure 3:**
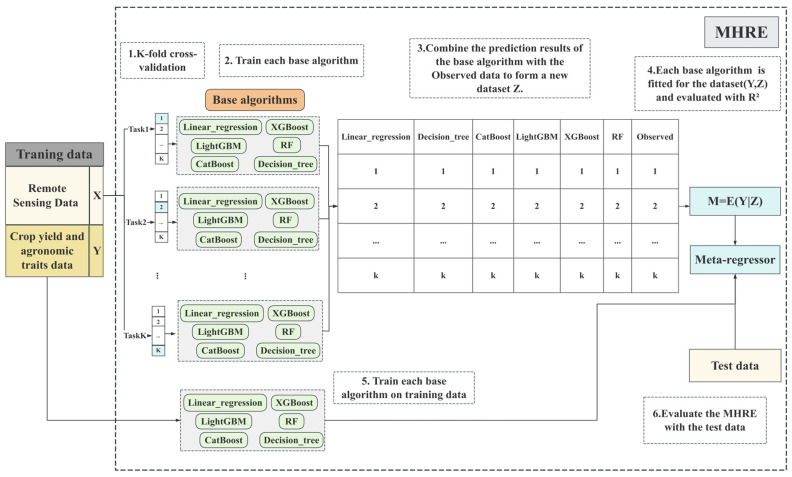
Technical modeling diagram for the MHRE framework, M denotes the meta-learner that combines the predictions of the six base models.

The MHRE (Meta-Hybrid Regression Ensemble) combined with six commonly used machine learning (ML) algorithms (Linear regression, Decision tree, LightGBM, RF, XGBoost, and CatBoost) is proposed for multi-source crop traits prediction (Figure 3). In order to avoid problems due to scale differences between the input features and the target variables, this study used normalization techniques and processing steps consistent with related studies. Optimal hyperparameters such as complexity, structure, and learning rate were tuned for each regression algorithm through grid search and k-fold cross-validation [50].

The algorithm constructs better predictive models by combining the predictions of several underlying ML algorithms [51]. Specifically, each base model is first trained on the original training data and then used to generate out-of-sample predictions on a held-out validation set; these predictions, together with the dependent variable Y, form the meta-dataset (Z, Y), where Z is the vector of base-model outputs. The performance of each base model is then evaluated on this meta-dataset using R2, and the best-performing model is chosen as the meta-regressor [52], which learns an optimized weighted or nonlinear combination of the base-model predictions to produce the final forecast. MHRE learns from a population of N independent samples (Yi, Xi) [53] through a two-stage training process: in the first stage, the dataset is randomly split into training and test sets, and the training set is further partitioned into K folds for cross-validation to generate the meta-dataset—each fold uses K − 1 subsets to train the six base models and the remaining subset to obtain out-of-sample predictions; the meta-regressor is then selected based on its R2 performance on this meta-dataset. In the second stage, all six base models are retrained on the entire training set, and for each new sample their predictions form the input vector Z to the already-selected meta-regressor, which produces the final prediction. Finally, the overall predictive performance of MHRE was evaluated on an independent test set.

### 2.5. Interpretability Analysis of the Model

To address the need for interpretability of ensemble ML models, this study adopts the SHapley additivity interpretation (SHAP) framework based on cooperative game theory. The SHAP technique, which originates from game theory, aims to improve the interpretability of ensemble ML and DL models by quantifying the contribution of each feature to the prediction. It evaluates the marginal contribution of each feature by comparing the prediction results with and without a feature, and the calculated Shapley value reveals the importance of individual features and their interactions. This approach provides insights that help to understand the decision-making process of complex models, thus making the models transparent and mining the relationships hidden in the data. We computed the shap values for each base learner, then we computed the shap values for the meta-learner, and finally we used a weighted synthesis approach to obtain the shap values for each feature [54]. See Equation (5) for more details.

(5) $$Final\text{\_}{values}_{i}=\sum_{j=1}^{n}w_{j}{\mathrm{SHAP}}_{B_{j}}(x_{i})$$

where $\mathrm{Final}\text{\_}{\mathrm{values}}_{i}$ denotes the contribution of the ith feature of the final synthesis. ${SHAP}_{B_{j}}\left(x_{i}\right)$ denotes the SHAP value of the jth base learner (e.g., Decision Tree, XGBoost, CatBoost, etc.) for the i-th feature. $w_{j}$ is the weight of the jth base learner in the meta-learner. n is the number of base learners.

### 2.6. Assessment of Predictive Performance

In this study, four indicators are mainly used to evaluate the model performance, namely, the R^2^, RMSE, RRMSE, MAE, and RPD. These metrics can fully reflect the prediction accuracy and error of the model [55]. Additionally, Pearson’s correlation coefficient was employed to assess the linear relationship between crop traits. Its use was justified as the data met the key assumptions of normality and linearity, as confirmed by Shapiro–Wilk tests and visual inspection of scatterplots [56]. The R^2^ (Equation (6)) is a statistic that measures the degree of fit between the predicted and actual values of a model, with values ranging from 0 to 1. The closer the value is to 1, the better the model explains the data.

(6) $$R^{2}=1-\frac{\sum_{i=1}^{n}{\left(y_{i}-{\hat{y}}_{i}\right)}^{2}}{\sum_{i=1}^{n}{\left(y_{i}-\overset{¯}{y}\right)}^{2}}$$

RMSE (Equation (7)) is a measure of the standard deviation of the difference between predicted and actual values; the smaller the value of RMSE, the smaller the model’s prediction error.

(7) $$\mathrm{RMSE}=\sqrt{\frac{1}{n}\sum_{i=1}^{n}{\left(y_{i}-{\hat{y}}_{i}\right)}^{2}}$$

RRMSE (Equation (7)), expressed as a percentage, is derived by normalizing the RMSE [57]. This normalization provides an intuitive measure of the error magnitude relative to the average level of the observed values, and lower RRMSE values correspond to better model performance.

(8) $$RRMSE=RMSE/\overset{¯}{y}\times 100\%$$

MAE (Equation (9)) quantifies model performance by calculating the average of the absolute errors between predicted and actual values [58]. Smaller MAE values indicate higher predictive accuracy and provide a robust measure of the typical prediction error.

(9) $$\mathrm{MAE}=\frac{1}{n}\sum_{i=1}^{n}\left|y_{i}-{\hat{y}}_{i}\right|$$

In addition, the ratio of performance to deviation (Equation (10)) was used as a dimensionless indicator to compare the predictive ability of different models, and higher RPD values indicate better predictive performance.

(10) $$RPD=\frac{{SD}_{obs}}{RMSE}$$

In all of the above equations, $y_{i}$ and ${\hat{y}}_{i}$ denote the observed and predicted values of the target trait for sample i, $\overset{¯}{y}$ is the mean of the observed values, n is the number of samples, and ${\mathrm{SD}}_{\mathrm{obs}}$ is the standard deviation of the observed values in the validation set.

## 3. Results

### 3.1. Overall Performance in Major Crop Traits Prediction

#### 3.1.1. Prediction of Major Traits of Rice

Table 3 systematically illustrates the performance of XGBoost, RF, LightGBM, CatBoost, and MHRE models in the prediction of crop growth parameters in the experimental field. The results demonstrate that the MHRE model has superior performance in the prediction of rice yield and related agronomic traits. Particularly, in the ES prediction task, MHRE and XGBoost achieved the same performance in terms of R^2^ (0.64). However, MHRE achieved a lower RMSE (27.81 10,000·spike ha^−1^), RRMSE (11.10%), RPD(2), and MAE (21.79 10,000·spike ha^−1^), indicating its superior capability in error control. In general, although the base models achieved a certain level of accuracy in predicting yield and other key agronomic traits, RF demonstrated relatively stable performance in some metrics due to its random sampling and feature-splitting mechanisms, but its overall predictive accuracy was still lower than that of the MHRE model.

**Figure 4:**
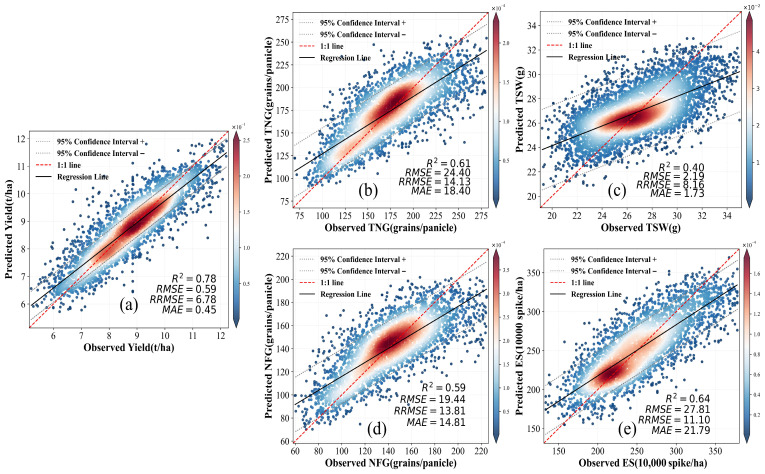
Accuracy of the model for the prediction of rice traits. (**a**) regression analysis of predicted and measured Yield; (**b**) Total number of grains (TNG); (**c**) Thousand seed weight (TSW); (**d**) Number of filled grains (NFG); and (**e**) Effective spikes (ES).

In yield prediction, the MHRE model achieved the optimal prediction accuracy (R^2^ = 0.78, RMSE = 0.59 t ha^−1^) (Figure 4a), and its performance was significantly better than the benchmark model. The model also showed a gradient performance characteristics for the prediction of other key traits: the highest prediction accuracy was achieved for the ES, R^2^ = 0.64 (Figure 4e), followed by TNG, R^2^ = 0.61 (Figure 4b) and the NFG, R^2^ = 0.59 (Figure 4d), while the prediction of the TSW was relatively low in terms of explanatory power (R^2^ = 0.40) (Figure 4b), and its prediction results exhibited a higher degree of uncertainty. The results demonstrate that meta-learning-based stacked integration strategy effectively improves the prediction robustness of complex agronomic traits under the condition of heterogeneous data from multiple sources and provides reliable technical support for crop growth monitoring in precision agriculture.

#### 3.1.2. Prediction of Major Cotton Traits

Comparative analysis shows that the MHRE model outperforms single algorithms in predicting major cotton traits (Table 4). In particular, for stem height prediction, the MHRE framework achieved the same coefficient of determination (R^2^ = 0.80) as the RF model but showed higher precision with a lower RMSE (7.72 cm), RRMSE (7.06%), and MAE (5.79 cm). This performance advantage is particularly pronounced for complex, yield-related parameters, suggesting that MHRE captures more effectively the intricate relationships between explanatory variables and phenotypic outcomes. In contrast, traditional XGBoost, CatBoost, and LightGBM models performed relatively worse on the same dataset, although they provided decent predictions in some cases.

**Figure 5:**
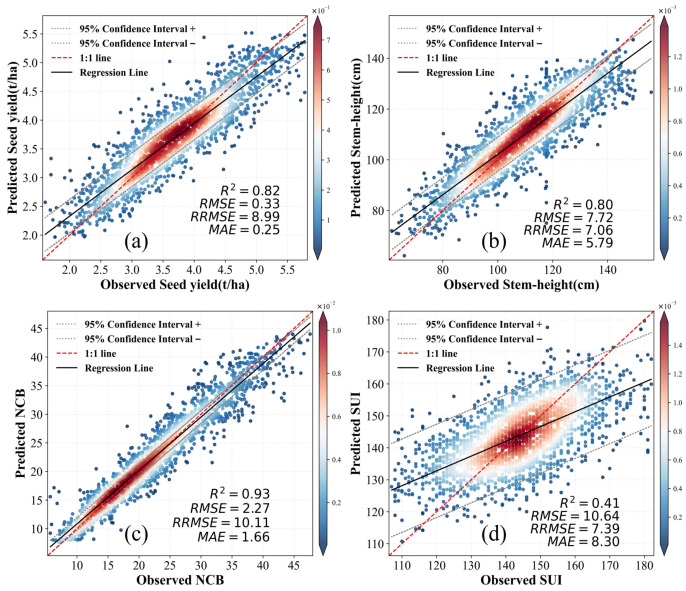
Accuracy of the model for the prediction of major cotton traits. (**a**) regression analysis of predicted and measured Seed-yield values; (**b**) Stem-height; (**c**) Number of bolls per plant (NCB); (**d**) Spinning uniformity index (SUI).

Experimental validation on an independent test set confirmed the model’s robust predictive ability across multiple key traits. In holdout trial predictions, several traits reached an R^2^ of 0.41 or higher. The prediction accuracy for Seed-yield reached R^2^ = 0.82 (Figure 5a). The model also excelled at structural traits, including NCB (R^2^ = 0.93; Figure 5c) and stem height (R^2^ = 0.80; Figure 5b), effectively capturing key features closely related to plant growth structure. Remarkably, MHRE also retained predictive capability for the SUI (R^2^ = 0.41; Figure 5d)—a composite metric reflecting fiber spinnability and estimated yarn strength. Although the dispersion in SUI predictions suggests residual variation likely due to the trait’s inherent complexity, the ensemble approach still outperformed all individual methods across every evaluated trait. These results demonstrate that ML ensemble strategies are highly effective in addressing the complex phenotype-environment relationships in agricultural crop traits prediction.

### 3.2. Robustness of Variety-Specific Trait Predictions via Stratified Validation

#### 3.2.1. Robustness of Trait Prediction Across Rice Varieties

**Figure 6:**
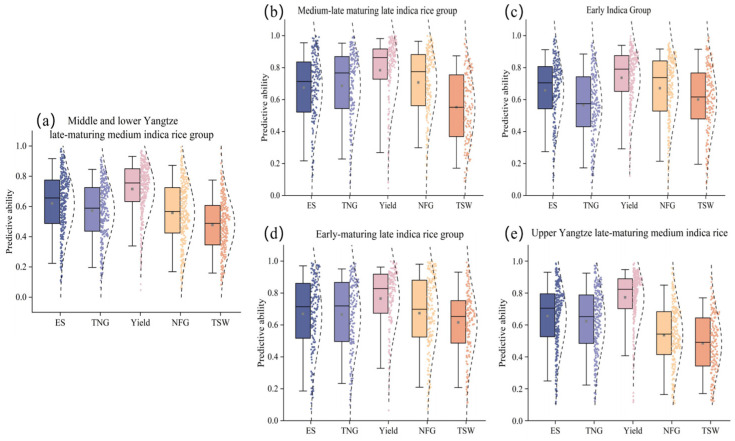
Predictive stability of different rice varieties. (**a**) Middle and lower Yangtze River late-maturing medium-indica, (**b**) Medium-indica late-maturing, (**c**) Early indica group, (**d**) Early-maturing late-indica, and (**e**) Upper Yangtze River late-maturing medium-indica.

To validate the predictive ability of the model across different rice varieties, we adopted a stratified leave-one-out cross-validation strategy, using the coefficient of determination (R^2^) as the evaluation metric. To rigorously prevent data leakage—particularly temporal bias that could arise from having the same growing year represented in both training and validation sets—the stratification was carefully designed to ensure that all data from a specific sub-variety and growing year were kept within the same fold and exclusively assigned to either training or validation in each iteration. Figure 6 shows the predictive ability of the traits for five types of rice: late-maturing medium-indica in the middle and lower reaches of the Yangtze River (Figure 6a), late-maturing medium-indica in the upper reaches of the Yangtze River (Figure 6e), late-maturing medium-indica (Figure 6b), early-maturing late-indica (Figure 6d), and the early indica group (Figure 6c). Under stratified leave-one-out cross-validation, we predicted the yield and other agronomic traits for each sub-variety within every rice variety and calculated the corresponding prediction accuracy. Each scatter point represents the prediction accuracy for a specific sub-variety. The study found that the middle and lower reaches of the Yangtze River (Figure 6a) and the upper reaches (Figure 6e) of late-maturing medium-yielding indica varieties showed similar prediction patterns, with excellent stability in yield prediction (average R^2^ > 0.7). In contrast, the NFG and TSW showed a wider dispersion and significant clustering of outliers in these groups. All the variety groups had the common feature of weak explanatory power for TSW. In particular, the two late-indica subgroups—late-maturing (Figure 6b) and early-maturing (Figure 6d)—both achieved excellent yield prediction accuracy (R^2^ > 0.8). However, the prediction ranges for ES and TNG were limited, while NFG and TSW still showed considerable variation. The early indica group (Figure 6c) presented a particular challenge, with significant variation in the prediction of TNG and TSW. The MHRE model maintained consistent prediction accuracy for major rice traits under variety-specific growth patterns, highlighting the robustness of the model in dealing with the phenotypic complexity of the rice system.

#### 3.2.2. Robustness of Trait Prediction Across Cotton Varieties

**Figure 7:**
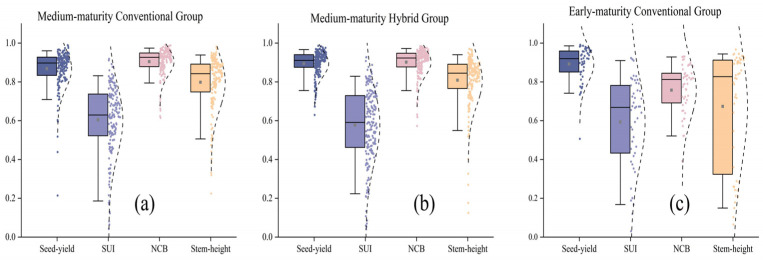
Predictive stability of different cotton varieties. (**a**) Medium-maturing conventional varieties, (**b**) Medium-maturity hybrids, and (**c**) Early maturity routine.

The generalizability of the model was further confirmed through a systematic evaluation of three cotton cultivars: medium-maturing conventional cotton (Figure 7a), medium-maturing hybrid cotton (Figure 7b), and early-maturing conventional cotton (Figure 7c), with all evaluations using the same validation scheme. It is noteworthy that both medium-maturing cultivar groups showed robustness in predicting key traits: Seed-yield (medium-maturing conventional: R^2^ = 0.84, medium-maturing hybrid = 0.89), NCB (medium-maturing conventional: 0.86, medium-maturing hybrid: 0.90), and Stem-height (medium-maturing conventional: 0.74, medium-maturing hybrid: 0.81), with data points densely distributed within the 95% confidence interval. The hybrid cultivar (Figure 7b) showed higher prediction consistency, particularly for seed yield. In contrast, the early-maturing conventional cotton group (Figure 7c) exhibited trait-specific prediction differences. Although seed yield (mean R^2^ = 0.85) and number of bolls per plant (0.76) maintained high prediction accuracy, plant height prediction showed significant fluctuations (IQR = 0.57). In particular, the SUI presented the greatest prediction challenge across all cultivars.

### 3.3. Spatial Applicability Under Geographically Stratified Validation

#### 3.3.1. Spatial Applicability for Major Rice Traits

**Figure 8:**
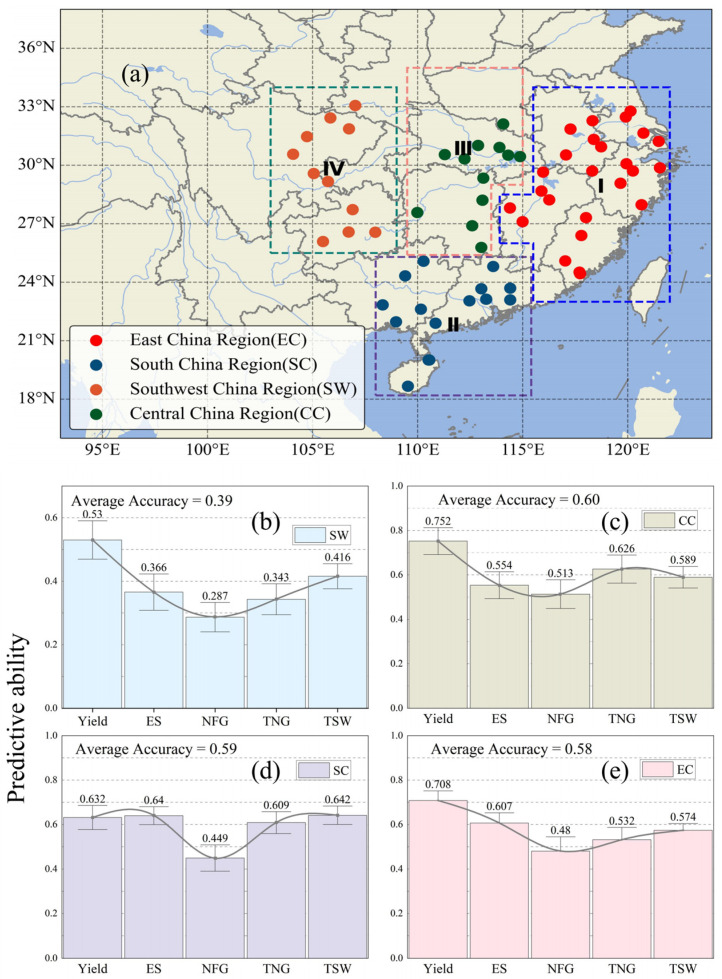
Bar charts of the study regions and the predictive power of the model. (**a**) The geographic locations of different regions in China, including South China (SC), Southwest China (SW), East China (EC), and Central China (CC). (**b**) Predictive ability for different traits in SW. (**c**) Predictive ability for different traits in CC. (**d**) Predictive ability for different traits in SC. (**e**) Predictive ability for yield and other traits in EC.

To rigorously evaluate the spatial generalization ability of the model (Figure 8), we constructed a geographically stratified validation framework covering the four major rice production regions in China: East China (EC), South China (SC), Central China (CC), and Southwest China (SW). In the evaluation process, R^2^ was used as the primary performance metric to quantify the model’s predictive accuracy across different regions. A systematic geographically stratified leave-site cross-validation method was applied using all observation stations within each region as independent test sets (EC: 24 stations, CC: 12 stations, SC: 12 stations, SW: 11 stations). This approach ensured the comprehensiveness and reliability of the evaluation, allowing for an assessment of the model’s performance across different regional environmental conditions. In particular, Central China (CC) showed the best prediction performance, with an overall accuracy index of 0.60 (Figure 8c), and an average R^2^ of 0.752 for yield-related traits. East China and South China maintained robust predictive ability, with average accuracy indices of 0.58 (Figure 8e) and 0.59 (Figure 8d). By contrast, Southwest China exhibited a lower average accuracy (Figure 8b), which is likely related to its complex topography and highly fragmented rice-growing landscapes, where small paddy fields are frequently mixed with forests, built-up areas and other land uses at the 1 km scale. This spatial heterogeneity, together with variable climate and management and fewer field trial sites, reduces the effective signal-to-noise ratio and limits prediction accuracy in this region.

#### 3.3.2. Spatial Applicability for Major Cotton Traits

**Figure 9:**
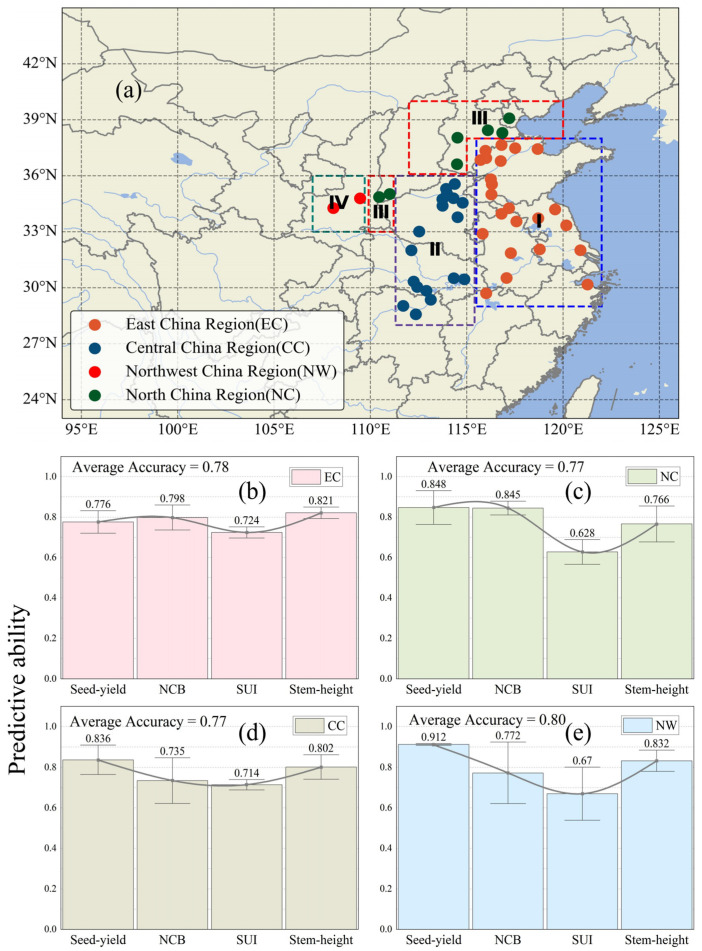
Bar charts showing the study regions and the predictive power of the model. (**a**) shows the geographic locations of different regions in China, including North China (NC), Northwest China (NW), East China (EC), Central China (CC), and South China. (**b**) shows the predictive ability for different traits in EC, (**c**) in NC, (**d**) in CC, and (**e**) in NW.

To rigorously assess the spatial transferability of the model (Figure 9), we developed a geographically stratified validation framework covering the four major cotton production regions in China: EC, North China (NC), CC, and Northwest China (NW). A station-by-station iterative validation protocol was implemented at all monitoring sites (EC: n = 23, NC: n = 7, CC: n = 17, and NW: n = 2), systematically quantifying the cross-regional prediction performance.

The overall average accuracy indices for the four major macro-regions were: East China (EC) 0.78 (Figure 9b), North China (NC) 0.77 (Figure 9c), Central China (CC) 0.77 (Figure 9d), and Northwest China (NW) 0.80 (Figure 9e). Levene’s test showed no significant differences in prediction performance for major cotton traits across regions: the *p*-value for NCB was 0.53, for seed yield 0.57, for SUI 0.81, and for stem height 0.61, all >0.05. Notably, East China showed higher consistency in trait prediction accuracy, with the best match between predicted and observed values. It is particularly noteworthy that the Spinning Uniformity Index (SUI) consistently proved to be the most challenging prediction target, with significantly lower accuracy compared to other traits. This performance pattern remained consistent across different geographical layers, indicating the inherent complexity in modeling this fiber quality parameter.

### 3.4. SHAP Framework for Evaluating Key Factors in the Yield Formation Process of Rice and Cotton

**Figure 10:**
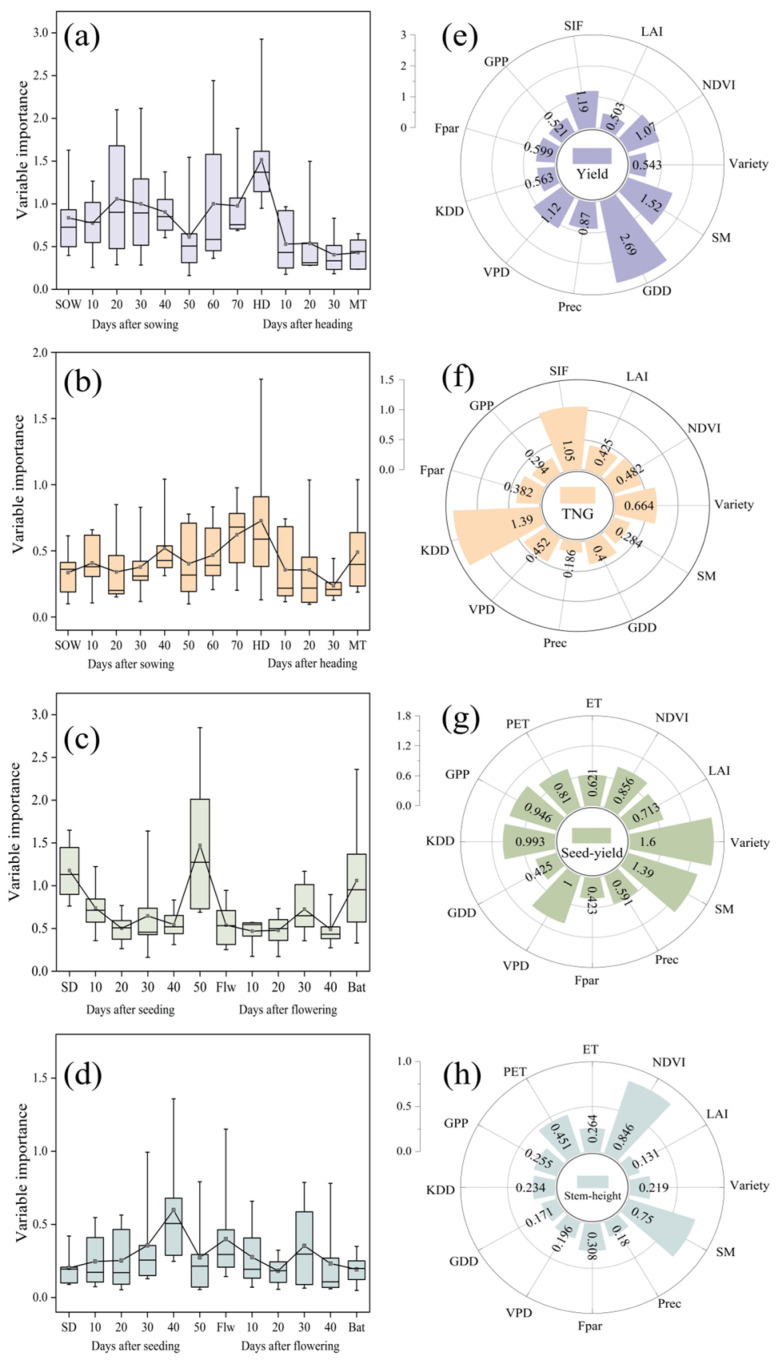
Variable importance over time and combined contribution of variables. (**a**) Importance of variables at different days after sowing and after heading for rice yield, (**b**) after sowing and after heading for rice TNG, (**c**) after seeding and after flowering for cotton seed yield, (**d**) after sowing and after heading for cotton stem height (**e**) Combined importance of variables for rice yield, (**f**) for TNG, (**g**) for cotton seed yield and (**h**) for cotton stem height.

This study employs the SHAP interpretability framework to systematically quantify the contributions of climatic and remote-sensing variables to both yield and other key crop traits (cotton stem height and rice total grain number, TNG) at critical growth stages. For rice (Figure 10a), yield exhibits a secondary sensitivity peak 20 days after sowing (SHAP = 1.10) and reaches its maximum influence at the heading stage (SHAP = 1.51). TNG (Figure 10b) is most responsive during heading stage (SHAP = 0.73). Multi-dimensional analysis (Figure 10e) identifies GDD (SHAP = 2.69), SM (SHAP = 1.52), and SIF (SHAP = 1.19) as the principal drivers of rice yield, while TNG (Figure 10f) is chiefly influenced by KDD (SHAP = 1.39) and SIF (SHAP = 1.05). As shown in Figure 10c, the seeding stage exhibited relatively high sensitivity (SHAP > 1.10), peaking at 50 days after seeding (SHAP = 1.47). Stem-height analysis (Figure 10d) indicates that internode elongation is most sensitive around 40 days after seeding. Overall (Figure 10g), SM (SHAP = 1.39), varietal genetic characteristics (SHAP = 1.60), and vapor pressure deficit (VPD; SHAP = 1.00) dominate cotton yield formation, whereas stem height (Figure 10h) is primarily regulated by NDVI (SHAP = 0.85) and SM (SHAP = 0.75). These distinct spatiotemporal sensitivity patterns—cotton’s mid-growth peak affecting boll and stem development versus rice’s sowing and heading stages—reveal crop-specific resource-allocation strategies, where temperature and canopy photosynthesis around heading govern panicle formation, spikelet fertility and grain filling in rice, and water availability and evaporative demand during flowering and batting strongly regulate boll retention in cotton. This alignment between SHAP-derived sensitivities and established physiological understanding provides a basis for precisely targeting management to the most influential growth stages and for optimizing both yield and other key traits.

## 4. Discussion

### 4.1. Potential of MHRE in Major Crop Traits Prediction

Our research shows that the MHRE model effectively integrates satellite remote sensing, meteorological data, and crop growth data to successfully predict major crop traits for different varieties and ecological zones. This is particularly valuable in addressing issues commonly encountered in traditional methods, such as observation gaps, spatial sampling bias, and high data collection costs, thereby demonstrating the significant value of low-resolution satellite data [59]. Compared to traditional ML algorithms, the MHRE model, by integrating multiple suboptimal algorithms, is able to extract complex features from high-dimensional data, significantly improving prediction accuracy. In addition to R^2^- and error-based metrics, the RPD values further support the advantage of MHRE over the individual base learners. For most key traits, MHRE achieves the highest RPD in Table 3 and Table 4, indicating better discrimination between signal and noise and consistent superiority across multiple performance criteria. While UAV-based methods achieve high trait-prediction accuracy [60], their limited spatial coverage impedes large-scale application. Similarly, regression models integrating ground and multispectral data perform well locally but fail in heterogeneous landscapes due to static observations [61]. Deep learning approaches face computational and data-quality constraints despite strong performance on complex traits [62]. In contrast, MHRE overcomes these limitations by leveraging low-resolution satellite data. The MHRE model has shown stability and consistency across different varieties and geographic regions, integrating multidimensional information from diverse areas to reveal the complex interactions between environmental and genetic factors. Despite robust performance across most regions, MHRE faced accuracy challenges in topographically complex areas like the SW (Figure 8c) due to landscape fragmentation [63]. Additionally, prediction accuracy varies across different traits. For example, ES (Figure 4e), which is highly correlated with canopy spectral features, shows higher prediction accuracy, whereas TSW (Figure 4c), influenced by genetic traits and environmental interactions, has a lower prediction accuracy [64]. SHAP value analysis reveals different physiological drivers in the rice and cotton systems. SHAP analysis not only supports the biological plausibility of the model but also provides novel insights into crop response mechanisms by quantifying the contributions of environmental drivers across different growth stages and traits [65]. Temporal SHAP profiling reveals sensitive windows beyond the reproductive phase that may have received comparatively less emphasis in prior analyses. For example, a distinct secondary sensitivity peak in rice yield occurs at 20 days after sowing (SHAP = 1.10), preceding the main peak at the heading stage (SHAP = 1.51). Notably, this early peak reaches ~73% of the heading peak (1.10/1.51), indicating a non-trivial early-season constraint that would be understated by conventional stage-averaged interpretations. This indicates that early vegetative conditions may exert a lasting effect on final yield formation—a temporal nuance that may be underrepresented in conventional growth models [66]. Meanwhile, the responses of different traits to environmental drivers show clear differentiation. In rice, TNG is primarily influenced by heat stress (KDD, SHAP = 1.39) and canopy photosynthetic capacity (SIF, SHAP = 1.05) [67], whereas accumulated thermal time (GDD, SHAP = 2.69) contributes more strongly to overall yield. This provides a quantitative constraint hierarchy: for TNG, KDD exceeds SIF by 0.34 SHAP units (~32% higher), while for yield, GDD shows a markedly larger contribution (2.69), suggesting distinct physiological “control points” for yield components versus final yield. This suggests that yield components are governed by distinct physiological pathways [68]. In cotton, stem height is jointly influenced by canopy greenness (NDVI, SHAP = 0.85) [69] and soil moisture (SM, SHAP = 0.75), reflecting tight coupling between canopy status and water availability during vegetative growth [70]. In contrast, seed yield is regulated by an integration of varietal traits, SM, and VPD, highlighting the composite nature of reproductive success [71,72]. Furthermore, the concurrent elevation of SHAP values for multiple variables during key periods implies potential synergistic stress effects. For instance, whole-season contribution analysis (Figure 10g) identifies SM and VPD as dominant factors for cotton yield, while temporal dynamic analysis (Figure 10c) reveals a broadly sensitive window around mid-season (approximately 50 days after seeding). This pattern is consistent with a compound water-stress period as flowering approaches, during which limited soil water supply and high atmospheric vapor pressure deficit may jointly intensify plant water stress and thereby increase the risk of boll shedding—an effect that may be difficult to capture in single-factor study designs [73]. Importantly, the SHAP framework provides quantifiable and comparable measures of influence, moving beyond qualitative descriptions such as “water is important” or “heat stress is critical.” For example, for TNG of rice, the SHAP value for KDD (1.39) is quantitatively higher than that for SIF (1.05), indicating that under the observed conditions, heat stress imposed a stronger limitation on grain number than photosynthetic capacity. By translating physiological narratives into comparable effect sizes, the model outputs yield directly testable priorities: which driver matters more, for which trait, and at which time window. These data-driven findings offer a mechanistic and quantitative basis for targeting management practices to specific developmental windows and for prioritizing stress-resilience traits in breeding programs within the complex context of genotype–environment–management interactions.

In this study, SHAP is used as a model-based attribution approach to summarize how the fitted predictor-to-trait mapping distributes importance across drivers, traits, and time, rather than as a tool for causal mechanism discovery. Compared with correlation-based summaries, temporal SHAP profiles provide a stage-resolved view of attribution, helping to localize periods when the model is most sensitive to specific drivers (e.g., early-season and mid-season windows) that are not directly captured by season-averaged analyses. Compared with standard sensitivity/importance summaries that provide a single global ranking, temporal SHAP additionally provides stage-resolved attribution patterns. In addition, because SHAP values are reported on a comparable contribution scale within the fitted model, they enable quantitative, within-model comparisons among drivers for a given trait and stage (e.g., KDD vs. SIF for rice TNG under the observed conditions), complementing qualitative interpretation. We note important limitations: SHAP explanations are model-dependent and do not establish causality; moreover, when predictors are correlated, attribution can be non-unique. Accordingly, the physiological interpretations are framed as testable, attribution-consistent hypotheses to be evaluated against established experimental and modeling evidence.

MHRE takes the variety encoding (as a proxy for genotype), environmental variables, and stage-specific remote-sensing–derived phenotypic indicators as joint inputs. The base learners comprise both linear and nonlinear models. In particular, nonlinear learners such as tree ensembles and gradient boosting can learn conditional response patterns, under which the contribution of an environmental factor may vary with genotype and environmental context, whereas the linear learner mainly provides a relatively stable characterization of global main effects. The meta-learner then integrates complementary patterns learned by different base learners, thereby better representing stage-dependent and nonlinear response differences. Accordingly, the G × E effects referred to in this study are implicitly characterized through the joint inclusion of genotype-proxy and environmental predictors together with the ensemble’s capacity to represent nonlinear/interaction-like responses, rather than through an explicit decomposition of variance into G, E, and G × E components.

Correlation between yield and other crop traits. Pearson analyses (Table 5 and Table 6) highlight distinct yield–trait linkages in the two cropping systems. Pearson’s correlation was applied to evaluate linear associations between cotton traits, as the data conformed to bivariate normality assumptions, verified by Shapiro–Wilk tests and Q-Q plots. Scatterplots confirmed linearity, satisfying the method’s key requirements. Rice yield showed a moderate, statistically significant association with both NFG (r = 0.52, *p* < 0.05) and TNG (r = 0.44, *p* < 0.05). These reproductive components, therefore, appear to be the primary drivers of yield variability within our multi-regional data set. In contrast, TSW is only weakly correlated with yield (r = 0.19), and ES shows essentially no linear relationship (r = −0.08). Specifically, NFG and TNG are themselves highly collinear (r = 0.87, *p* < 0.05), whereas ES is negatively related to both NFG and TNG (each r = −0.54, *p* < 0.05), reflecting the well-known trade-off between panicle number and grain set. Cotton seed yield correlated most strongly with the NCB (r = 0.32, *p* < 0.05). Relationships between SUI (r = 0.15) and stem height (r = 0.18) were weak and not statistically significant, indicating that fiber quality and vegetative vigor do not materially constrain yield in the current germplasm set. A moderate positive correlation between NCB and stem height (r = 0.55, *p* < 0.05) suggests that taller plants tend to carry more fruiting sites, but this structural trait did not translate directly into higher lint yield. Overall, the correlation patterns corroborate the MHRE model outputs: traits with the greatest physiological leverage on yield (NFG and TNG in rice; NCB in cotton) coincide with those traits for which the model achieved the highest prediction accuracies (Figure 4).

In addition, the predictive performance across traits should be interpreted in light of both their variability and the extent to which that variability is captured by the predictors. Descriptive statistics (Table 7) show that some traits have much larger Standard Deviation (SD) and Coefficient of Variation (CV) than others. For rice, TSW exhibits a comparatively modest R^2^ (0.40) despite its observed variability, suggesting that part of its variation is driven by heterogeneous field conditions and measurement noise that are not fully represented by the predictors. For cotton, NCB combines high variability with very high R^2^, indicating that most of its dispersion is aligned with the remote-sensing and meteorological predictors. However, the SUI shows low variability but only modest R^2^ (0.41), suggesting that much of its remaining variation reflects fiber-quality genetics and measurement noise beyond the environmental predictors. These examples show that large variability can lower R^2^ when it is mainly driven by noise or unobserved factors, whereas traits whose variability is well explained by the available predictors can still be predicted accurately even when their dispersion is large.

### 4.2. Potential Limitations

Although the MHRE model demonstrates superior predictive performance compared to traditional ML models, three key limitations need to be addressed. First, varietal-specific prediction differences reveal a model extrapolation bottleneck for modern breeding traits, which requires dynamic adaptation through the integration of genome-wide selection signals and phenotypic plasticity parameters [74]. While ML architectures have strong interpolation capabilities, they face limitations in extrapolating beyond the training domain, especially under novel environmental mechanisms [75]. MHRE represents G × E in an implicit, predictive manner and therefore cannot resolve several types of G × E complexity. Specifically, it does not explicitly partition variance into genotype (G), environment (E), and G × E components, and it does not provide explicit or definitive causal inference or mechanistic evidence. It has limited extrapolation to unseen genotypes because variety encoding is only a proxy, and it may not disentangle higher-order interactions (e.g., G × E × management) or rare genotype–environment combinations when management variables are unobserved or data coverage is sparse. In addition, fixed stages/windows may not fully capture genotype-dependent phenological shifts, which can blur genotype-specific environmental sensitivities. Second, insufficient representation of terrain-mediated resource heterogeneity (e.g., water redistribution patterns between plains and highlands) may affect the ability of models to transfer across terrain types [76]. Third, as the global climate becomes increasingly non-stationary [77,78], the predictability of precipitation patterns and temperature, particularly in China’s grain-growing regions, has decreased significantly [79], especially the non-stationarity of rainfall—about a quarter of the Earth’s land area now experiences non-stationary rainfall patterns [80], and this proportion is increasing. The non-stationarity of climate distribution and management practices introduces inherent uncertainty, and the combined effects of climate change and topographic diversity create sudden uncertainties in crop response trajectories, posing challenges for breeding target selection and trait prediction. Furthermore, although non-cropland pixels were removed using the national 1 km land-use dataset and local canopy conditions were represented by averaging cropland pixels within a 2 × 2 neighbourhood around each plot, the use of 1 km resolution remote-sensing data inevitably leads to residual mixed-pixel effects. This issue is particularly relevant in regions with complex topography and highly fragmented fields (e.g., parts of Southwest China), where individual pixels may still contain a mixture of crop and non-crop surfaces. Such pixel-scale heterogeneity can dilute crop-specific spectral signals and thus introduce additional uncertainty into the trait predictions, potentially contributing to the lower accuracy observed in these areas. Moreover, the spatial generalization demonstrated here should be understood as valid within the six studied production regions; applying MHRE to entirely new agro-ecological zones will require further local calibration and independent validation.

### 4.3. Future Enhancements

To further improve the predictive capability of agricultural modeling, we believe that efforts should be focused on three collaborative areas. First, a multi-dimensional integration framework should be established that combines crop genotype features with field management records to form a mechanistic model of genotype × environment × management (G × E × M) interactions [81] to improve biological realism in yield simulations. Second, adaptive data assimilation mechanisms should be developed to dynamically integrate multi-source observational data [82], including high-resolution satellite imagery, soil moisture sensor networks, and distributed meteorological stations, through self-optimization parameter calibration [83] to mitigate the effects of non-stationarity. Third, hybrid architectures combining process-based crop models with deep neural networks show great potential in balancing the predictive capabilities of data-driven models with the inherent physiological interpretability of mechanistic approaches. These collaborative strategies will collectively address the increasing environmental variability and severe food security challenges, making integrated crop models indispensable tools for crop breeding and agricultural planning. In addition, the current implementation of MHRE is crop-specific, with separate models trained for rice and for cotton. A promising direction for future work is to extend the framework towards a more unified multi-crop setting, for example, by adopting multi-task or hierarchical architectures that jointly model several crop species while retaining crop-specific responses. Such cross-crop modeling could better exploit shared patterns among crops and potentially improve trait prediction in data-scarce crops or regions, thereby further enhancing the generality and practical value of the MHRE framework.

## 5. Conclusions

This study proposed the MHRE framework, an innovative artificial intelligence approach that integrates multi-source data and interpretable modeling to significantly advance the prediction of major crop traits across diverse regions and crop varieties. By combining large-scale standardized datasets, multi-modal features, and robust validation, the framework demonstrated both high predictive performance and meaningful biological interpretability. Specifically, it achieved substantial improvements over baselines in predicting rice (R^2^ = 0.78, +6.85%; RMSE = 0.59 t·ha^−1^, −9.61%) and cotton (R^2^ = 0.82, +10.8%; RMSE = 0.33 t·ha^−1^, −14.1%) yields, alongside key traits like rice thousand seed weight (R^2^ +29%). Moreover, MHRE exhibits strong generalization, evidenced by robust performance in independent tests across six ecological zones (e.g., cotton R^2^ decline NW to NC: only 0.80 to 0.77, −3.8%). In terms of interpretability, this study reveals distinct spatiotemporal influence in yield and key traits (cotton stem height, rice TNG) to environmental and varietal drivers. In particular, it identifies divergent optimization phases: rice yield exhibits strongest driver responses at heading stage (driven by GDD and SIF), while cotton yield shows maximal effects mid-season (controlled by soil moisture and genetics). These insights establish a vital foundation for developing stage-specific crop management strategies. In conclusion, the MHRE framework demonstrates considerable advancements in both predictive accuracy and interpretability for major crop traits prediction. Its ability to achieve high performance across multiple varieties and ecological regions establishes it as a powerful tool for precision agriculture based on an artificial intelligence approach.

## Figures and Tables

**Table 1:** Details of the remote sensing datasets used in this study.

Data Sources	Date Type	Variables	Temporal Resolution	Spatial Resolution
MODIS	MOD13A1	NDVI	16 days	500 m
	MCD15A3H	LAI	4 days	500 m
	MCD15A3H	Fpar	4 days	500 m
	MOD16A2	ET	8 days	500 m
	MOD16A2	PET	8 days	500 m
	MOD17A2H	GPP	8 days	500 m
TROPOMI	RTSIF	SIF	8 days	0.05°
National Tibetan Plateau Data Center	SM	SM	1 day	1 km

**Table 2:** Parameters related to field trials in cotton and rice.

Crop	Data Types	Variable	Abbreviation
Rice	Phenological stages	Sowing date	Sow
		Heading date	HD
		Mature date	MT
		Growth duration	GD
	Rice agronomic traits	Yield (t ha^−1^)	-
		thousand seed weight (g)	TSW (g)
		Effective spike (10,000·spike ha^−1^)	ES (10,000·spike ha^−1^)
		Number of filled grains (grains/panicle)	NFG (grains/panicle)
		Total number of grains (grains/panicle)	TNG (grains/panicle)
Cotton	Phenological stages	Seeding date	SD
		Flowering date	Flw
		Batting date	Bat
		Growth duration	GD
	Cotton agronomic & fiber traits	Spinning uniformity index	SUI
		Stem-height (cm)	-
		Number of bolls per plant	NCB
		Seed-yield (t ha^−1^)	-

**Table 3:** Comparing the performance of five regression algorithms for the prediction of major rice traits.

Traits	Units	Regression Algorithm	R^2^	RMSE	RRMSE	MAE	RPD
Yield	t·ha^−1^	MHRE	**0.78**	**0.59**	**6.78**	**0.45**	**2.12**
		RF	0.69	0.70	7.98	0.64	2.05
		CatBoost	0.71	0.69	7.88	0.53	1.99
		XGBoost	0.76	0.61	6.99	0.46	1.82
		LightGBM	0.75	0.63	7.20	0.48	1.80
ES	10,000·spike·ha^−1^	MHRE	**0.64**	**27.81**	**11.10**	**21.79**	**2.02**
		RF	0.60	30.28	12.03	23.59	1.87
		CatBoost	0.63	30.87	12.15	24.05	1.85
		XGBoost	0.64	29.37	11.64	22.58	1.93
		LightGBM	0.63	29.55	11.76	22.96	1.91
TNG	grains/panicle	MHRE	**0.61**	**24.40**	**14.13**	**18.40**	**1.59**
		RF	0.58	27.11	15.52	19.95	1.45
		CatBoost	0.58	27.83	15.76	20.39	1.43
		XGBoost	0.59	27.03	15.52	19.63	1.45
		LightGBM	0.58	27.24	15.61	19.78	1.44
NFG	grains/panicle	MHRE	**0.59**	**19.44**	**13.81**	**14.81**	**1.57**
		RF	0.54	22.75	15.99	16.64	1.35
		CatBoost	0.56	21.77	15.36	16.19	1.41
		XGBoost	0.57	21.49	15.12	15.81	1.43
		LightGBM	0.56	22.06	15.51	16.17	1.40
TSW	g	MHRE	**0.40**	**2.19**	**8.16**	**1.73**	**1.29**
		RF	0.31	2.42	9.02	1.91	1.17
		CatBoost	0.31	2.37	8.80	1.88	1.19
		XGBoost	0.28	2.41	8.95	1.90	1.17
		LightGBM	0.32	2.34	8.69	1.88	1.21

**Bold** values indicate the best-performing model for each trait on the test set, considering R^2^ together with RMSE, RRMSE, MAE, RPD.

**Table 4:** Comparing the performance of five regression algorithms for the prediction of major cotton traits.

Traits	Units	Regression Algorithm	R^2^	RMSE	RRMSE	MAE	RPD
Seed-yield	t·ha^−1^	MHRE	**0.82**	**0.33**	**8.99**	**0.25**	**2.30**
		RF	0.77	0.36	9.69	0.28	2.13
		CatBoost	0.68	0.43	11.65	0.34	1.77
		XGBoost	0.76	0.38	10.17	0.29	2.03
		LightGBM	0.77	0.37	9.95	0.29	2.08
NCB	bolls·plant^−1^	MHRE	**0.93**	**2.27**	**10.11**	**1.66**	**3.79**
		RF	0.89	2.98	13.14	2.22	2.91
		CatBoost	0.87	3.20	14.02	2.37	2.73
		XGBoost	0.89	2.86	12.68	2.09	3.03
		LightGBM	0.89	2.84	12.62	2.08	3.02
SUI	index	MHRE	**0.41**	**10.64**	**7.39**	**8.30**	**1.30**
		RF	0.34	12.26	8.53	9.61	1.13
		CatBoost	0.40	11.84	8.23	9.25	1.17
		XGBoost	0.40	11.40	7.90	8.85	1.22
		LightGBM	0.40	11.57	8.04	9.08	1.20
Stem-height	cm	MHRE	**0.80**	**7.72**	**7.06**	**5.79**	**2.22**
		RF	0.80	7.97	8.57	7.44	1.83
		CatBoost	0.74	8.99	8.20	7.08	1.91
		XGBoost	0.79	8.05	7.40	6.22	2.12
		LightGBM	0.79	8.10	7.44	6.39	2.10

**Bold** values indicate the best-performing model for each trait on the test set, considering R^2^ together with RMSE, RRMSE, MAE, RPD.

**Table 5:** Pearson correlation analysis among rice traits.

Traits	Yield	ES	TSW	NFG	TNG
Yield	1				
ES	−0.08	1			
TSW	0.19	−0.30	1		
NFG	0.52 *	−0.54 *	−0.06	1	
TNG	0.44 *	−0.54 *	−0.03	0.87 *	1

* indicates significance at the 0.05 level (*p* < 0.05).

**Table 6:** Pearson correlation analysis among cotton traits.

Traits	Seed-Yield	NCB	SUI	Stem-Height
Seed-yield	1			
NCB	0.32 *	1		
SUI	0.15	0.13	1	
Stem-height	0.18	0.55 *	0.06	1

* indicates significance at the 0.05 level (*p* < 0.05).

**Table 7:** Descriptive statistics of observed rice and cotton traits used for model training and evaluation.

Crop	Trait	Unit	n	Mean	SD	Min	Max	CV (%)
Rice	Yield	t·ha^−1^	13,950	8.76	1.25	5.17	12.36	14.35
	ES	10,000·spike·ha^−1^	13,798	15.69	3.53	7.9	25.3	22.47
	TNG	grains/panicle	13,781	172.59	38.82	69.5	280.8	22.49
	NFG	grains/panicle	13,430	140.77	30.46	59.2	224.6	21.64
	TSW	g	13,935	26.89	2.83	19.0	35.0	10.51
Cotton	Seed-yield	t·ha^−1^	8026	3.71	0.77	1.61	5.80	20.68
	NCB	Bolls·plant^−1^	8016	22.55	8.63	5.1	47.6	38.27
	SUI	index	7862	144.33	13.91	107.0	182.0	9.63
	Stem-height	cm	8012	109.38	17.12	60.1	155.9	15.66

## Data Availability

The data are available on request.
